# Longitudinal development of the gut microbiome and metabolome in preterm neonates with late onset sepsis and healthy controls

**DOI:** 10.1186/s40168-017-0295-1

**Published:** 2017-07-12

**Authors:** Christopher J. Stewart, Nicholas D. Embleton, Emma C. L. Marrs, Daniel P. Smith, Tatiana Fofanova, Andrew Nelson, Tom Skeath, John D. Perry, Joseph F. Petrosino, Janet E. Berrington, Stephen P. Cummings

**Affiliations:** 1 Alkek Center for Metagenomics and Microbiome Research, Department of Molecular Virology and Microbiology, Baylor College of Medicine, Houston, Texas 77030 USA; 2 Newcastle Neonatal Service, Royal Victoria Infirmary, Newcastle upon Tyne, NE1 4LP.77030 UK; 3 Department of Microbiology, Freeman Hospital, Newcastle upon Tyne, NE7 7DN UK; 4 Faculty of Health and Life Sciences, Northumbria University, Newcastle upon Tyne, NE1 8ST UK; 5 School of Science and Engineering, Teesside University, Middlesbrough, TS1 3BX UK

**Keywords:** Preterm infant, Gut microbiome, Metabolomics, Late onset sepsis

## Abstract

**Background:**

Late onset sepsis (LOS) in preterm infants is associated with considerable morbidity and mortality. While studies have implicated gut bacteria in the aetiology of the disease, functional analysis and mechanistic insights are generally lacking. We performed temporal bacterial (*n* = 613) and metabolomic (*n* = 63) profiling on extensively sampled stool from 7 infants with LOS and 28 matched healthy (no LOS or NEC) controls.

**Results:**

The bacteria isolated in diagnostic blood culture usually corresponded to the dominant bacterial genera in the gut microbiome. Longitudinal changes were monitored based on preterm gut community types (PGCTs), where control infants had an increased number of PGCTs compared to LOS infants (*P* = 0.011). PGCT 6, characterised by Bifidobacteria dominance, was only present in control infants. Metabolite profiles differed between LOS and control infants at diagnosis and 7 days later, but not 7 days prior to diagnosis. Bifidobacteria was positively correlated with control metabolites, including raffinose, sucrose, and acetic acid.

**Conclusions:**

Using multi-omic analysis, we show that the gut microbiome is involved in the pathogenesis of LOS. While the causative agent of LOS varies, it is usually abundant in the gut. Bifidobacteria dominance was associated with control infants, and the presence of this organism may directly protect, or act as a marker for protection, against gut epithelial translocation. While the metabolomic data is preliminary, the findings support that gut development and protection in preterm infants is associated with increased in prebiotic oligosaccharides (e.g. raffinose) and the growth of beneficial bacteria (e.g. *Bifidobacterium*).

**Electronic supplementary material:**

The online version of this article (doi:10.1186/s40168-017-0295-1) contains supplementary material, which is available to authorized users.

## Background

Late onset sepsis (LOS; defined as sepsis after 72 h of life) remains a serious and common complication of prematurity, with rates of 20–40% for infants <32 weeks gestation reported in some studies. LOS in preterm infants impacts negatively on survival (with mortality rates of up to 10%) and on developmental outcomes [1]. Mechanisms of LOS pathogenesis are poorly understood, but bacterial colonisation and low gestational age are key risk factors [2]. Bacterial profiling studies have shown that LOS infants have an altered microbiome and lower bacterial diversity [3–8], and the bacterial strain isolated in diagnostic blood culture is frequently present in the gut [9]. Central to LOS pathogenesis are bacterial-host interactions modulating gut and systemic immune responses, tight junction integrity, and host metabolic function [10]. The most common organisms causing LOS in preterm infants include coagulase-negative *Staphylococcus*, *Escherichia*, *Klebsiella*, and *Enterococcus* [11].

Recent advances in ultra-performance liquid chromatography-mass spectrometry (UPLC-MS) untargeted metabolomics facilitate further understanding of these complex relationships involving host and bacteria, and the complex interactions of immune and metabolic function in relation to health and disease states [12]. While largely pilot in nature, existing metabolomic studies in preterm infants have demonstrated important findings. In necrotizing enterocolitis (NEC), the most prevalent serious preterm disease after LOS, the metabolite profiles are different at diagnosis compared to controls in serum [13–15], urine [16], and stool [17]. Stool volatile organic compound and serum UPLC-MS has also demonstrated differences between LOS infants and matched controls at or immediately prior to disease [13, 15, 18]. Stool metabolite profiles are also significantly associated with age [19] and serum metabolite profiles between preterm and term neonates also differ [14].

We aimed to explore relationships between gut microbiome and metabolome to determine key insights into LOS development, impact, and recovery. This is the first study to employ UPLC-MS untargeted metabolomics of stool to determine host and bacterial functioning within the gut of infants diagnosed with LOS.

## Results

### Infant and sample information

We recruited a large cohort of preterm infants, sampling stool daily where possible (*n* = >300 infants/>3000 samples) and capturing key health-related outcomes using precise definitions [17, 20, 21]. Using strict classification for LOS (positive blood culture with a >5 days antibiotics) and only including infants with robust temporal sampling before and after disease diagnosis, we present comprehensive longitudinal gut microbiome data on 613 stool samples from LOS infants (*n* = 7) and well-matched non-diseased (no NEC or LOS) controls (*n* = 28). A subset of 63 stool samples from LOS infants (*n* = 4) and matched controls (*n* = 10) also underwent UPLC-MS.

Infant demographics are shown in Table 1 and further detail provided in Additional file 1: Table S1. The average number of samples for LOS and control infants was comparable (15 vs. 18, respectively). Infant demographics were comparable between LOS and matched controls, although as expected, antibiotic use was increased in LOS (antibiotic information provided in Additional file 2: Table S2). Diagnostic blood culture identified two cases of *Staphylococcus aureus*, two cases of *Staphylococcus epidermidis*, one case of *Enterococcus faecalis*, one case of *Streptococcus agalactiae*, and one case of *Escherichia coli* (Additional file 1: Table S1).

**Table 1:** Summary of infant samples and demographic per group

	Control (*n* = 28)	LOS (*n* = 7)	*P* value
Number of stool samples	520	106	–
Gestation (weeks)*	27 (25–28)	27 (26.5–28)	0.393
Birth weight (g)*	910 (863–1199)	1000 (725–1105)	0.612
Birth mode (CS/vaginal)	12/16	3/4	1.0
Gender (male/female)	20/8	4/3	0.475
Breast milk exposure (yes/no)	25/3	7/0	0.820
Antibiotic prediagnosis (days)*	–	7 (2–14)	–
Antibiotic total (days)*	4.5 (2–9.5)	23 (10–32)	0.071

*Median (interquartile range)

### The abundant bacterial genus in the gut microbiome preceding diagnosis corresponds to the genera of the causative agent in LOS

**Fig. 1:**
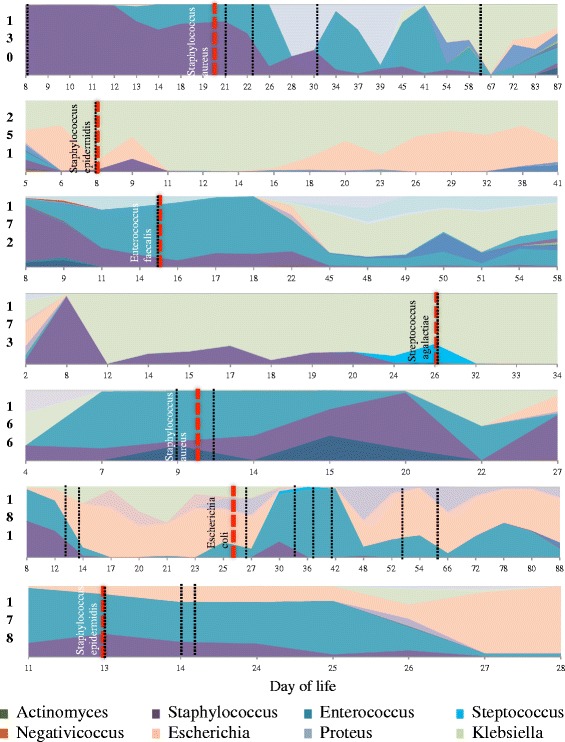
Area plots showing the temporal development of the microbiome in infants diagnosed with late onset sepsis (*LOS*). *Dashed red lines* represent the day of LOS diagnosis with the bacteria isolated from blood culture identified. *Dashed black lines* represent the start of an antibiotic treatment as per Additional file 2: Table S2.

The gut microbiome of infants with LOS were highly individual and dynamic through time. The pathogen identified by blood culture was one of the most abundant OTUs in the gut microbiota at diagnosis, with the corresponding genus from the gut microbiome the most abundant in four cases and second most abundant OTU in two cases (Fig. 1). An exception was *Staphylococcus epidermidis* LOS in infant 251, which was the seventh most abundant genus at diagnosis. In all cases, the genera of the bacteria isolated in diagnostic blood culture were present prior to LOS diagnosis. For infant 173 who was diagnosed with *S. agalactiae*, the organism was detected 2 days before diagnosis and within 6 days of antibiotic treatment (flucloxacillin and gentamicin) it was no longer detected.

### Preterm gut community types dominant in Bifidobacterium are protective for LOS

**Fig. 2:**
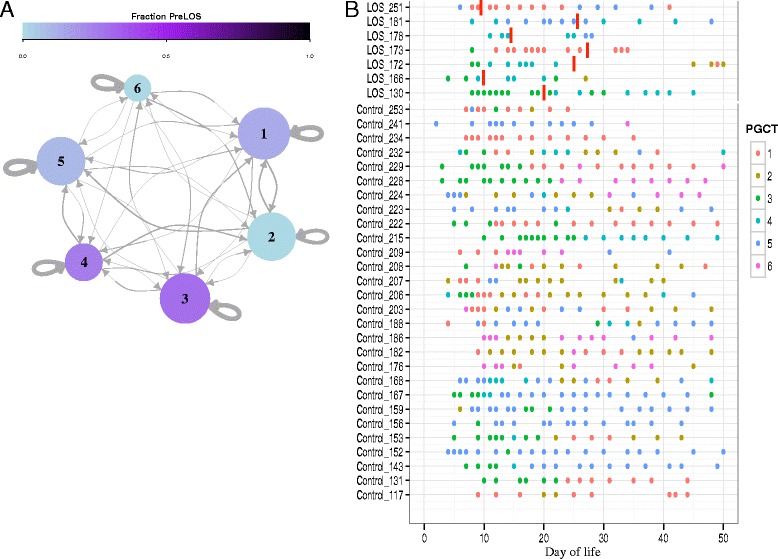
Characterisation of the gut microbiome between infants diagnosed with late onset sepsis (*LOS*) and matched controls. **a** Transition network analysis showing PGCTs in PreLOS samples compared to matched controls approximated as a Markov chain with subject-independent transition probabilities. *Arrow weights* reflect the transition probabilities from one sample to the next. Size of *circle* reflects the relative number of samples associated with that PGCT. *Pale blue* indicates PGCTs of consisting of control samples only, and the *darker shade of purple* shows increased number of PreLOS samples in that PGCT. **b** Temporal change in PGCTs in each individual infant. *Red lines* represent day of LOS diagnosis. Only samples up to day 50 of life are included. Infant 178 died during the study

To further explore the complexity in the developing preterm microbiome, we employed PAM clustering analysis to ascertain preterm gut community types (PGCTs), as previously described [17]. All samples grouped into six discrete clusters (Additional file 3: Figure S1): dominance of *Klebsiella* (PGCT 1), dominance of both *Klebsiella* and *Enterococcus* (PGCT 2), dominance of *Staphylococcus* (PGCT 3), dominance of *Enterococcus* (PGCT 4), dominance of *Escherichia* (PGCT 5), and mixed population with high relative abundance of *Bifidobacterium* (PGCT 6) (Additional file 4: Figure S2). No PGCT was strongly associated with PreLOS samples when compared to all control samples, whereas PGCT 2 and PGCT 6 were never found in any sample from LOS infants before diagnosis (Fig. 2a). PGCT 6 was also never found in any LOS infant after diagnosis, and PGCT 2 was present in only 2 infants after diagnosis, detected >2 weeks following diagnosis and treatment (Fig. 2b and Additional file 4: Figure S2). Conversely PGCT 6, which represents a diverse community high in relative *Bifidobacterium* abundance, was present frequently throughout the control population from early to late samples. Specifically, PGCT 6 was detected in 65 samples from 12 control infants, representing 43% of the control population. Counting the number of unique PGCTs over the first 25 days of life showed control infants had an average of 3 unique PGCTs, compared to an average of 2 unique PGCTs in preLOS samples (*P* = 0.011).

### Untargeted metabolomic profiling indicates distinct functional profiles between infants with LOS and controls

**Fig. 3:**
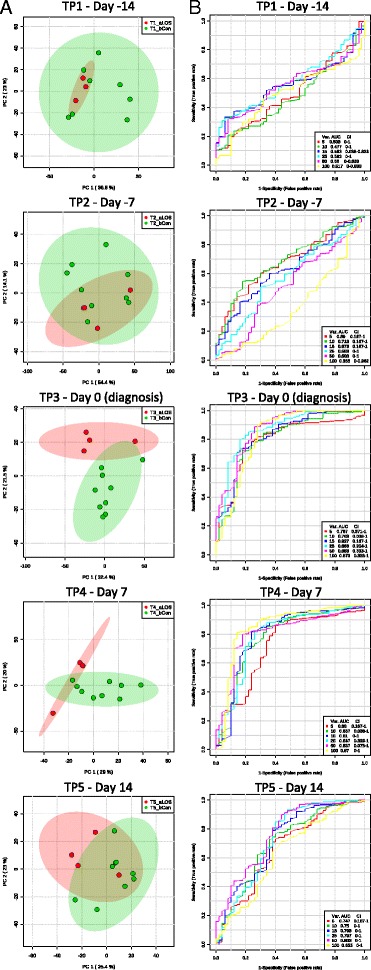
Metabolomic profiles between infants diagnosed with LOS and matched controls across all 5 time points, where TP3 represents samples at diagnosis. **a** PCA (unconstrained ordination) of LOS infants (*red*) and matched controls (*green*). Each sample represented by the *small circle* and *ellipses* represent the 95% confidence interval. **b** Receiver operating characteristic curves of support vector machine predictions for LOS and control samples. AUC represents the strength of the predictive classifications. Selected number of metabolites computed in intervals from 5, 10, 15, 25, 50, and 100 metabolites

Given the finding that the dominant genus in the microbiome is associated with the causative agent in LOS, we further investigated the potential functional differences in the gut between infants diagnosed with LOS and matched controls. Due to sample availability, this pilot experiment included 4 infants with LOS (infants 130, 172, 181, and 251) and 10 matched controls, across 5 time points spanning before and after LOS diagnosis: −14 days (time point 1; TP1), −7 days (TP2), 0 days (TP3), +7 days (TP4), and +14 days (TP3), relative to diagnosis of LOS. Although each LOS infant had different bacterial species isolated in diagnostic blood culture (Fig. 1), PCA showed metabolite profiles clustering distinctly between LOS infants and matched controls, with the most profound differences at diagnosis (0 days) and +7 days (Fig. 3). Receivers operating characteristic (ROC) curves were generated, and area under a ROC curve (AUC) was implemented to provide a measure of how well metabolites distinguish between LOS and matched controls. Lines progressing towards the upper-left corner of plots represent better discrimination (higher sensitivity and specificity). At diagnosis, the AUC ranged from 0.787 with 5 metabolites to 0.883 with 25 metabolites and both equated to a sensitivity of 75% and a specificity of 89%.

**Fig. 4:**
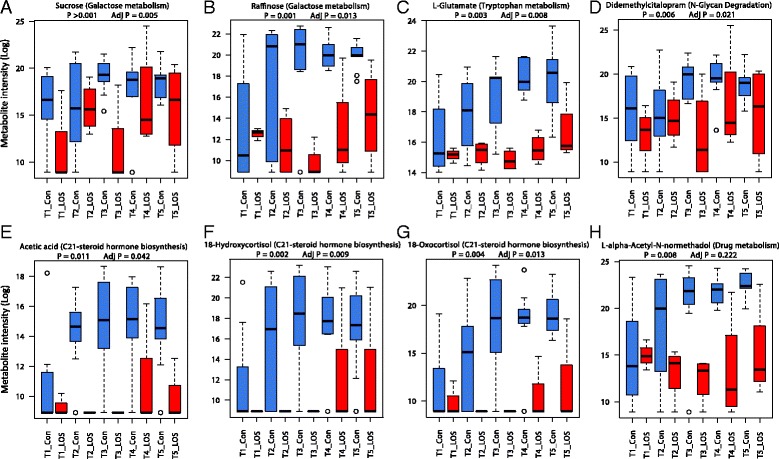
Box plots to show the levels of significant metabolites though each time point between infants diagnosed with late onset sepsis (LOS) and matched controls. Plots listed in order of significance. **a** Sucrose. **b** Raffinose. **c** L-Glutamate. **d** Didemethylcitalopram. **e** Acetic acid. **f** 18-Hydroxycortisol. **g** 18-Oxocortisol. **h** L-alpha-Acetyl-N-normethadol

Fourteen stool metabolites were identified as significantly altered between LOS and control infants at diagnosis (TP3), with 7 metabolites (all increased in controls) remaining significant following adjustment for 7 confounders (Table 2). Galactose metabolism was the most frequently increased pathway in control infants, and sucrose (*P* = 0.001) and raffinose (*P* = 0.001), both from galactose metabolism, were the most significant metabolites. These metabolites increased through time within control infants, whereas the same metabolites remained at baseline or reduced prior to diagnosis in LOS infants (Fig. 4). Notably, following diagnosis and treatment, these metabolites increased in LOS infants, but tended to remain at lower intensity compared to controls (with the exception of metabolites from C21-steroid hormone biosynthesis that remained at baseline throughout). Taken together, the PCA and box plot analysis of the most significant metabolites suggest altered and delayed functional development in the gut in LOS infants prior to diagnosis.

**Table 2:** List of metabolites and pathways significantly altered between control and LOS infants at diagnosis (day 0)

	Metabolite	Pathway	Fold change	Log2(FC)	*P* value	Adjusted *P* value
Increased in controls	Sucrose	Galactose metabolism	15.6	3.96	0.001	0.005
	Raffinose	Galactose metabolism	1963.1	10.94	0.001	0.014
	18-Hydroxycortisol	C21-steroid hormone biosynthesis and metabolism	10683	13.38	0.003	0.010
	L-Glutamate	Tryptophan metabolism	29.33	4.87	0.003	0.008
	18-Oxocortisol	C21-steroid hormone biosynthesis and metabolism	17551	14.10	0.005	0.013
	Didemethylcitalopram	N-Glycan degradation	6.14	2.62	0.007	0.021
	L-alpha-Acetyl-N-normethadol	Drug metabolism-cytochrome P450	676.77	9.40	0.009	0.226
	Acetic acid	C21-steroid hormone biosynthesis and metabolism	577.12	9.17	0.011	0.042
	Lactose	Galactose metabolism	11.66	3.54	0.033	0.123
	3-Ketolactose	Galactose metabolism	555.27	9.12	0.047	0.002
Increased in LOS	21-Hydroxy-5beta-pregnane-3,11,20-trione	C21-steroid hormone biosynthesis and metabolism	0.004	−7.93	0.034	0.137
	10,11-dihydro-leukotriene B4	Leukotriene metabolism	0.16	−2.68	0.034	0.153
	Monoethylglycinexylidide	Drug metabolism-cytochrome P450	0.29	−1.79	0.039	0.125
	11-Deoxycortisol	C21-steroid hormone biosynthesis and metabolism	0.004	−7.93	0.043	0.147

Abbreviations: *LOS* late onset sepsis, *FC* fold change

### Multi-omic analysis shows distinct correlations between significant metabolites and abundant bacterial genera

**Fig. 5:**
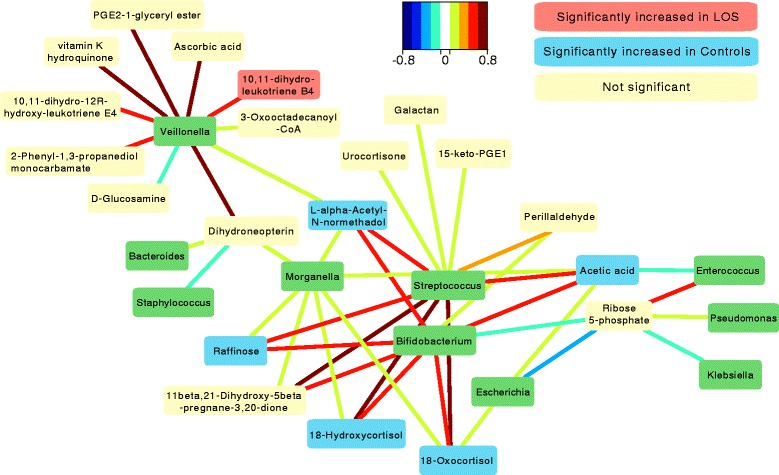
Spares partial least squared correlations (sPLS) between dominant bacterial genera and identified metabolites. sPLS in regression mode (predict Y from X) to model a causal relationship between bacterial genera and metabolites. Bacterial genera represented in *green* boxes. *Red* boxes are metabolites significantly increased in LOS, *blue* boxed significantly increased in controls, *yellow* boxes are not significantly altered between LOS and controls. Significant metabolites based on the samples at diagnosis (time point 0)

sPLS correlation analysis was performed using MixOmics to determine the correlations between the dominant bacterial genera and identified metabolites (Fig. 5). *Bifidobacterium* and *Streptococcus* showed comparable strong positive correlations with a range of metabolites that were significantly increased in control infants (Table 2), including raffinose, 18-hydroxycortisol, 18-oxocortisol, acetic acid, and L-alpha-acetyl-N-normethadol. These findings were supported when only including control infants in the analysis; demonstrating diagnosis is not confounding the results (Additional file 5: Figure S3). Furthermore, this correlation was lost when analysing a shuffled dataset and is thus not an artifact of autocorrelation. *Morganella* also showed weak positive correlation with these metabolites. *Veillonella* showed distinct clustering with strong correlations to a range of metabolites, including vitamin K and ascorbic acid (vitamin C), as well as 10,11-dihydro-12R-hydroxy-leukotriene E4 that was significantly increased (*P* = 0.032) in LOS infants. *Staphylococcus*, *Bacteroides*, *Escherichia*, *Klebsiella*, *Enterococcus*, and *Pseudomonas* all showed weak correlations with the detected metabolites.

## Discussion

We explored gut microbiome and metabolome factors that are altered in the development of LOS in preterm infants and show that the dominant taxa in the gut microbiome are usually isolated in diagnostic blood culture. Control infants had greater microbiome development and prevalence of PGCT 6 (*Bifidobacterium* dominant). Novel untargeted stool metabolomics on a subset of samples showed that metabolite profiles are significantly different between LOS and control infants at diagnosis and 7 days later. Stool metabolites from a range of pathways/sources, especially sucrose and raffinose, tended to increase in controls through time compared to LOS infants, where they remained low prior to and at diagnosis. Finally, combining omic datasets to explore correlations between the microbiome and metabolome showed *Bifidobacterium* was positively correlated with metabolites significantly increased in control infants.

The gut microbiome has been previously implicated in the pathogenesis of LOS [3–9], with whole genome shotgun sequencing revealing the exact strain in diagnostic blood culture can be detected in stool [9]. Our data concurs, showing the species cultured from blood was typically abundant from birth and corresponds to the most or second most abundant OTU in the gut at diagnosis. One exception occurred where the microbiome was dominated by *Klebsiella* and *Escherichia*, but *S. epidermidis* was isolated in blood culture. Due to the prevalence of *S. epidermidis* on the skin, this organism is a common contaminant of blood cultures, however, it is also commonly associated with preterm sepsis [22]. It is possible that different mechanisms are in action where LOS results from organisms that typically colonise the skin, for example, through compromised skin barrier from venepuncture, heel pricks etc., than where the mechanism is of translocation of gut bacteria [23].

PGCT 6 (Bifidobacteria dominant) was only found in control infants, detected in nearly half of all control infants (43%), but whether this is a cause of gut health or a marker of gut health is unknown. The potential health-promoting properties of *Bifidobacterium* are well reported [24–29]. Although the largest existing probiotic trial of 1315 preterm infants found no significant improvement to NEC or LOS rates using *Bifidobacterium breve* [30], only specific species of *Bifidobacterium* in the preterm gut are able to utilise human milk oligosaccharides (HMOs): these species appear to have key roles in establishing the pioneering species of the gut [31]. While *B. breve* produce fucosidases and sialidases, only *Bifidobacterium longum* subspecies *infantis* are able to digest all HMO structures [32]. The differences between timing, dose, and most importantly the *Bifidobacterium* species/strains used are likely to account for the inconsistent health benefits between *Bifidobacterium* probiotic studies. In a separate cohort, we have previously shown that *Bifidobacterium bifidum* deliberately administered to preterm infants can colonise and persist in threefold greater relative abundance following discharge, compared to control infants [33].

Metabolomics was employed to determine if the changes in gut microbiome were reflected at the functional level and if metabolic markers for LOS could be detected. LOS samples grouped distinctly at diagnosis (sensitivity of 75% and a specificity of 89%). Previous work has shown serum metabolite profiles [13] and stool volatile organic compound profiles [18] altered between LOS infants and controls within 3 days of onset, but no single metabolite being diagnostic of LOS. We found no differences in stool metabolites 14 or 7 days before diagnosis. Although significant in the unadjusted models, following adjustment for potential confounders, no metabolite was significantly increased in all LOS infants. This suggests the pathogenesis is of acute onset, with multiple aetiological components affecting individual infants differently.

Network analysis was employed to determine correlations between the microbiome and metabolome, revealing that *Bifidobacterium* and *Streptococcus* have comparable positive correlations. Given *Bifidobacterium* dominant communities were specific to control infants and *Bifidobacterium* is strongly associated with a healthy mature gut microbiome [24–29], it is notable that metabolites correlated with this genera were significantly increased in control infants. Metabolites correlated with *Bifidobacterium* and *Streptococcus* were from a range of pathways: raffinose (Galactose metabolism), L-alpha-acetyl-N-normethadol (Drug metabolism), and acetic acid, 18-hydroxycortisol, and 18-oxocortisol (C21-steroid hormone biosynthesis and metabolism). Raffinose is a derivative of sucrose, and these two metabolites were the most significant overall, with both increased in controls. Raffinose is a α-galactosyl (α-GAL) oligosaccharide, and because humans do not possess the α-GAL enzyme, it is fermented in the gut by bacteria containing the α-GAL enzyme. This metabolite reduced in LOS infants prior to diagnosis, increasing after treatment, whereas it remained consistently high in controls from day 7 (TP2). Raffinose is considered a prebiotic [34] that has been shown to inhibit the growth of potentially pathogenic bacteria [35] and to promote *Bifidobacterium* spp. in human and animal studies [36]. In addition, raffinose increases short chain fatty acid (SCFA) concentrations, specifically acetic and propionic acid, contributing to increased weight gain in animal models [37, 38] and a reduction in pathogenic bacteria [39]. Due to the use of LCMS, SCFA were not detected in the current study, but further work exploring the exact strains of *Bifidobacterium* and their specific effects on the gut microbiome and metabolome is warranted.

The study has several potential limitations. The strict inclusion criteria requiring extensive longitudinal sampling before and after disease diagnosis meant only 7 infants with LOS were included, despite collection of samples from >300 infants. However, this cohort size is comparable to existing studies in LOS and the findings here support existing data [3–7, 9]. Cost and sample size considerations meant metabolomics was performed on only four infants with LOS and ten controls and thus conclusions drawn this analysis should be considered preliminary. It is necessary to repeat the correlation analysis at specific time windows in larger cohorts. Nonetheless, this study has yielded important findings that warrant validation in large multi-center studies with extensive longitudinal sampling, particularly within 7 days of LOS diagnosis.

## Conclusions

Using novel multi-omic analysis, we show for the first time that the gut microbiome and metabolome are associated with the pathogenesis of LOS. In accordance with published data, we find the causative agent in LOS is usually abundant in the gut microbiome, suggesting translocation though the gut epithelium. This is further supported by the change in bacterial and host metabolism in the gut, which is reflective of altered function. While the causative agent of LOS varies, Bifidobacteria dominant communities were only found in controls and this taxa was further correlated with the metabolites significantly associated with control infants, including raffinose, sucrose, and acetic acid. The current study supports that gut development and protection in preterm infants is associated with increased in prebiotic oligosaccharides (e.g. raffinose) and the growth of beneficial bacteria (e.g. *Bifidobacterium*). The finding in the current study requires validation in a larger cohort and the exact mechanisms, and the development of therapies aimed at promoting health for preterm infants, such as pre- and probiotics, warrant further investigation.

## Methods

### Participants and study design

The study design, setting, participants, and methods of data collection have been reported previously [17, 40]. Briefly, all infants were cared for in a single unit with standardised feeding, antibiotic, and antifungal guidelines. LOS was defined as a positive blood culture treated with antibiotics for a minimum of 5 days along with signs consistent with sepsis reviewed independently by two clinicians. Cultured isolates from positive blood culture were identified using matrix-assisted laser desorption ionisation–time of flight mass spectrometry. All demographic information is summarised in Table 1, and full information for each infant is provided in Additional file 1: Table S1.

Stool samples and clinical data were collected from a total of 318 preterm infants at study conception. Seven well-sampled cases of LOS and 28 matched controls, free of LOS, or NEC, were selected based on extensive longitudinal sampling and matched by gestational age (GA; +/−1 week), birth weight, and delivery mode. A total of 613 analysed stool samples underwent 16S rRNA gene bacterial profiling. Metabolomic profiling was performed on a subset of 14 infants (63 stools): 4 LOS and 10 matched controls. LOS samples were selected for analysis relative to disease diagnosis at day of life (DOL) −14 (time point 1; TP1), −7 (TP2), 0 (TP3), +7 (TP4), and +14 (TP5), and controls were matched to this by DOL.

### 16S rRNA gene bacterial profiling

Nucleic acid extraction of stool was carried out on 100 mg of sample using the PowerLyzer™ PowerSoil® DNA Isolation Kit (MoBio, CA, USA) in accordance with the manufacturer’s instructions. Bacterial profiling utilised the 16S rRNA gene targeting variable region 4 based on the Schloss wet-lab MiSeq SOP and resulting raw fastq data were processed using Mothur (version 1.31.2), as described previously [41]. Briefly, combined reads were trimmed to 275 reads with 0 ambiguous bases. Chimeric sequences were detected by Chimera.uchime and were removed from downstream analysis. Alignment was generated via the Silva v4 database [42] and chloroplast, mitochondria, unknown, archaea, and eukaryota linages were removed from the analysis. Raw sequences were deposited in MG-RAST under the accession numbers 4516545.3-4516585.3.

### UPLC-MS metabolomic profiling

Metabolomic profiling was performed as previously described [19, 43]. Briefly, 100 mg stool was homogenised (80% methanol), was vortexed for 15 min, centrifuged (10000×g), and was lyophilised. Reverse-phase ultra-performance LCMS tandem mass-spectrometry (UPLC-MS/MS) was performed using an Accucore C18 column (2.6 μm, 150 × 2.1 mm) at 40 °C, 3.0 μl injection, and 300 μl/min flow rate. Gradients increased from 5% acetronitrile (ACN) to 95% ACN over 22 min, followed by 8 min wash, and re-equilibration. Samples were run randomly in triplicate on a Q-Exactive (Thermo) using HESI with high resolution (70,000) positive and negative switching. The mass range was set from 100–1000 m/z. SIEVE (version 2.2) was used to process the Thermo RAW files by component extraction.

### Bioinformatic and statistical analysis

#### Bacterial community analysis

16S bacterial profiles were analysed using a stand-alone tool for analysing and visualising microbiome data sets developed at the Center for Metagenomics and Microbiome Research at Baylor College of Medicine (not published), conducted in R version 3.3 [44]. Each sample was rarefied to 4397 reads. PGCTs were determined using a publically available script for linear mixed-effects modelling, medoid-based clustering, and Markov chain modelling [45]. Bray-Curtis was used to calculate the distance between all samples, and this was denoised by extraction of the most significant Principal Coordinates Analysis (PCoA) eigenvectors before applying the PAM algorithm. Gap statistic was used to determine the number of clusters. Significance of categorical variables was determined using the non-parametric Mann-Whitney test for comparison of LOS and control infants. Only taxa present in >1% relative abundance were included in statistical analysis. All *P* values were adjusted for multiple comparisons with the false discovery rate (FDR) algorithm [46].

#### Metabolomics analysis

UPLC-MS data was filtered to include only *m*/*z* features that occurred in >20% of samples. Metabolite annotation and pathway enrichment was performed using Mummichog [47]. Mummichog was used to determine significant pathways between infants diagnosed with LOS and matched controls. Unlike the microbiome dataset, metabolomics was performed at five specific time windows relative to LOS onset, with matched day of life control samples. Analysis was therefore cross sectional within the specific time windows. Metabolomic MetaboAnalyst 3.0 [48] was employed to generate PCA plots and to determine the AUC between LOS and control infants at each time point. Receivers operating characteristic (ROC) curves were generated by linear support vector machine (SVM) classification with Monte-Carlo cross validation using balanced subsampling. In each Monte-Carlo cross validation, two thirds of the samples were used to examine the feature importance, and the classification model was validated using the one third of samples left out. Several iterations were performed to determine the optimal number of metabolites to predict MV use, with analysis based on 5, 10, 15, 25, 50, or 100 of the top metabolites based on the average importance. Two-tailed Welch’s *t* test (<2 variables) or ANOVA (>2 variables) were used to determine significant metabolites. Regression models adjusted for seven potential confounding variables: delivery mode, gestation age, sex, feed (received some maternal breast milk or formula only), number of days of antibiotics treatment, number of antibiotics used, and age. *P* values were adjusted for multiple comparisons using FDR [46].

#### Integrated analysis of microbiome and metabolomic datasets

MixOmics [49] was implemented in R to determine the correlation between the relative abundance of the dominant bacterial taxa from 16S rRNA gene sequencing and the intensity of metabolites of interest by sparse partial least squares regression (sPLS) [50].

## Additional files

Additional file 1: Table S1. Extensive demographic info (DOCX 22 kb)

Additional file 2: Antibiotic information (DOCX 18 kb)

Additional file 3: Table S2. Figure S1. Gap statistic showing justification for selecting 6 clusters (PDF 30 kb)

Additional file 4: Figure S2. Heatmaps of the most dominant bacterial genera per each preterm gut community type (PGCT). (A) PGCT 1. (B) PGCT 2. (C) PGCT 3. (D) PGCT 4. (E) PGCT 15. (F) PGCT 6. Genera shown in *bold* are dominant in that PGCT. (PDF 348 kb)

Additional file 5: Figure S3. Spares partial least squared correlations (sPLS) between dominant bacterial genera and identified metabolites from control infants only. Analysis excludes infants diagnosed with late onset sepsis. Only significant metabolites based on the samples at diagnosis (time point 0) and the top 10 most abundant bacterial taxa were included. (PDF 145 kb)

## Abbreviations

LOS: Late onset sepsis
NEC: Necrotising enterocolitis
NICU: Neonatal intensive care unit
PGCT: Preterm gut community type
UPLC-MS/MS: Ultra-performance liquid chromatography mass spectrometry tandem mass-spectrometry
