# Supplementary material for: Longitudinal development of the gut microbiome and metabolome in preterm neonates with late onset sepsis and healthy controls
Source: Microbiome. 2017 Jul 12;5:75. doi: 10.1186/s40168-017-0295-1 (PMC5508794; doi:10.1186/s40168-017-0295-1)
Supplement: Supplementary file 1 — Extensive demographic info (DOCX 22 kb) [file 40168_2017_295_MOESM1_ESM.docx]

**Additional file 1: Table S1 – Extensive demographic information per patient**

| **Patient** | **Status** | **Delivery Mode** | **Sex** | **Birth Weight** | **GA** | **DOL BC 1** | **Organism(s) BC1** | **DOL BC 2** | **Organism(s) BC2** | **EBM ever** | **DOL First EBM** | **DOL Full Feed 72hrs** | **DOL Last EBM** | **Antibiotics days** |
| --- | --- | --- | --- | --- | --- | --- | --- | --- | --- | --- | --- | --- | --- | --- |
| 130 | LOS | Caesarean | Male | 1000 | 27 | 20 | S.aureus |  |  | Yes | 4 | 15 | 89 | 36 |
| 166 | LOS | Vaginal | Male | 1250 | 29 | 10 | S.aureus |  |  | Yes | 4 | 22 | 6 | 17 |
| 172 | LOS | Vaginal | Female | 1060 | 28 | 15 | E.faecalis |  |  | Yes | 1 | 10 | Home breast fed | 9 |
| 173 | LOS | Caesarean | Male | 1150 | 28 | 27 | S.agalactiae group B |  |  | Yes | 2 | 18 | 22 | 13 |
| 178 | LOS | Caesarean | Female | 525 | 26 | 6 | S.epidermidis | 13 | S.epidermidis | Yes | 3 | Never/died | 28 | 33 |
| 181 | LOS | Vaginal | Female | 570 | 23 | 26 | E.coli |  |  | Yes | 4 | 14 | 86 | 43 |
| 251 | LOS | Vaginal | Male | 880 | 27 | 8 | S.epidermidis |  |  | Yes | 4 | 14 | Discharge* | 9 |
| 117 | Control | Caesarean | Male | 870 | 26 |  |  |  |  | Yes | 2 | 10 | Discharge* | 12 |
| 131 | Control | Caesarean | Female | 545 | 27 |  |  |  |  | Yes | 4 | 27 | Discharge* | 2 |
| 143 | Control | Vaginal | Male | 620 | 24 |  |  |  |  | Yes | 4 | 17 | 89 | 7 |
| 152 | Control | Vaginal | Male | 800 | 25 |  |  |  |  | Yes | 1 | 12 | 72 | 4 |
| 153 | Control | Caesarean | Male | 1310 | 28 |  |  |  |  | Yes | 6 | 18 | Home breast fed | 2 |
| 156 | Control | Vaginal | Male | 1630 | 29 |  |  |  |  | Yes | 1 | 7 | Home breast fed | 2 |
| 159 | Control | Caesarean | Male | 1035 | 26 |  |  |  |  | Yes | 3 | 14 | 13 | 10 |
| 167 | Control | Caesarean | Male | 1290 | 27 |  |  |  |  | No | Never | 11 | Never | 4 |
| 168 | Control | Caesarean | Male | 1135 | 28 |  |  |  |  | Yes | 6 | 18 | Home breast fed | 8 |
| 176 | Control | Caesarean | Male | 880 | 26 |  |  |  |  | Yes | 1 | 11 | 11 | 2 |
| 182 | Control | Caesarean | Female | 895 | 26 |  |  |  |  | Yes | 1 | 11 | Home breast fed | 2 |
| 186 | Control | Caesarean | Female | 840 | 26 |  |  |  |  | Yes | 1 | 10 | 59 | 2 |
| 188 | Control | Vaginal | Female | 750 | 24 |  |  |  |  | Yes | 2 | 39 | 82 | 13 |
| 203 | Control | Vaginal | Male | 1130 | 26 |  |  |  |  | Yes | 1 | 15 | 74 | 5 |
| 206 | Control | Vaginal | Male | 1255 | 28 |  |  |  |  | Yes | 0 | 11 | Discharge* | 7 |
| 207 | Control | Caesarean | Male | 1580 | 29 |  |  |  |  | Yes | 2 | 12 | 13 | 5 |
| 208 | Control | Caesarean | Male | 950 | 29 |  |  |  |  | Yes | 11 | 22 | 12 | 15 |
| 209 | Control | Vaginal | Female | 1100 | 28 |  |  |  |  | Yes | 0 | 14 | 24 | 2 |
| 215 | Control | Vaginal (FD) | Male | 1180 | 27 |  |  |  |  | No | 7 | 20 | 7 | 7 |
| 222 | Control | Vaginal | Female | 620 | 24 |  |  |  |  | Yes | 3 | 21 | 86 | 15 |
| 223 | Control | Vaginal | Female | 885 | 25 |  |  |  |  | Yes | 5 | 15 | 120 | 8 |
| 224 | Control | Vaginal | Male | 1170 | 28 |  |  |  |  | Yes | 2 | 24 | 41 | 4 |
| 228 | Control | Vaginal (Breech) | Male | 910 | 25 |  |  |  |  | Yes | 4 | 14 | 125 | 15 |
| 229 | Control | Vaginal (Breech) | Female | 910 | 25 |  |  |  |  | Yes | 4 | 14 | Discharge* | 31 |
| 232 | Control | Vaginal | Male | 695 | 24 |  |  |  |  | Yes | 1 | 15 | 109 | 18 |
| 234 | Control | Caesarean | Male | 1810 | 30 |  |  |  |  | No | Never | 6 | Never | 2 |
| 241 | Control | Vaginal | Male | 1320 | 30 |  |  |  |  | Yes | 1 | 9 | Discharge* | 2 |
| 253 | Control | Vaginal | Male | 905 | 27 |  |  |  |  | Yes | 4 | 16 | Discharge* | 4 |

*Patients were reciving EBM at discharge but no follow-up data was collected

DOL - Day of life

BC - Blood culture

EBM - Expressed Breast Milk

NA - Not available
