# Supplementary material for: Longitudinal development of the gut microbiome and metabolome in preterm neonates with late onset sepsis and healthy controls
Source: Microbiome. 2017 Jul 12;5:75. doi: 10.1186/s40168-017-0295-1 (PMC5508794; doi:10.1186/s40168-017-0295-1)
Supplement: Supplementary file 2 — Antibiotic information (DOCX 18 kb) [file 40168_2017_295_MOESM2_ESM.docx]

**Additional file 2: Table S2 – Table of antibiotic information for patients with NEC and/or sepsis**

| Patient Number | Abx start (days) | Abx 1 (days of usage) | Abx 2 (days of usage) | Abx 3 (days of usage) | Abx 4 (days of usage) | Abx 5 (days of usage) |
| --- | --- | --- | --- | --- | --- | --- |
| 181 | 0 | P (2) | G (2) |  |  |  |
|  | 13 | V (3) |  |  |  |  |
|  | 14 | F (9) |  |  |  |  |
|  | 27 | T (1) | C (1) | V (2) | G (4) | Me (17) |
|  | 33 | G (3) |  |  |  |  |
|  | 37 | G (3) |  |  |  |  |
|  | 41 | G (3) |  |  |  |  |
|  | 53 | A (2) | F (2) | G (2) |  |  |
|  | 61 | A (2) | F (2) | G (2) |  |  |
| 178 | 0 | P (2) | G (2) |  |  |  |
|  | 6 | C (3) | V (2) |  |  |  |
|  | 13 | V (2) | C (5) |  |  |  |
|  | 14 | M (9) |  |  |  |  |
|  | 15 | L (14) |  |  |  |  |
|  | 29 | C (1) | M (1) | V (1) |  |  |
| 130 | 0 | P (2) | G (2) |  |  |  |
|  | 1 | A (7) | M (7) |  |  |  |
|  | 8 | V (3) | C (3) |  |  |  |
|  | 21 | V (2) | C (2) |  |  |  |
|  | 23 | F (14) |  |  |  |  |
|  | 31 | G (6) |  |  |  |  |
|  | 63 | A (2) | F (2) | G (2) |  |  |
| 251 | 0 | P (2) | G (2) |  |  |  |
|  | 8 | V (7) | C (3) |  |  |  |
| 172 | 0 | P (2) | G (2) |  |  |  |
|  | 15 | F (5) | G (5) | A (5) |  |  |
| 173 | 0 | P (5) | G (5) |  |  |  |
|  | 26 | F (2) | A (8) | G (2) |  |  |
| 166 | 0 | P (2) | G (2) |  |  |  |
|  | 9 | C (2) | V (2) |  |  |  |
|  | 11 | F (13) |  |  |  |  |

A – Amoxicillin, C – Ceftazidine, F – Flucloxacillin, G – Gentamicin, L – Linezolid, M – Metronidazole, Me – Meropenem, P – Penicillin, T – Tazocin, V - Vancomycin
