# Supplementary figures and images for: Longitudinal development of the gut microbiome and metabolome in preterm neonates with late onset sepsis and healthy controls
Source: Microbiome. 2017 Jul 12;5:75. doi: 10.1186/s40168-017-0295-1 (PMC5508794; doi:10.1186/s40168-017-0295-1)

Gap Statistic results

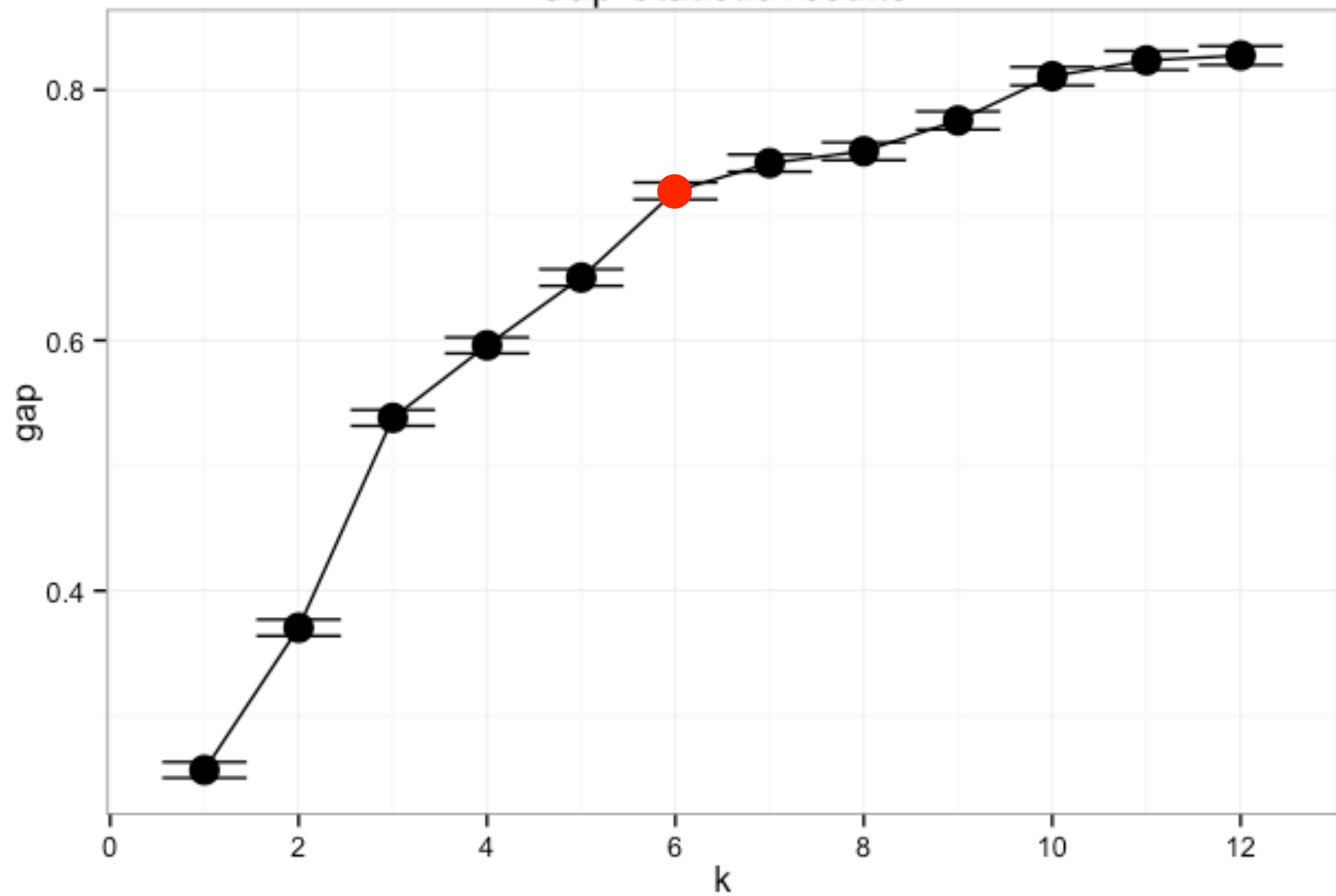

Supplement: Supplementary file 3 — Figure S1. Gap statistic showing justification for selecting 6 clusters (PDF 30 kb) [file 40168_2017_295_MOESM3_ESM.pdf]

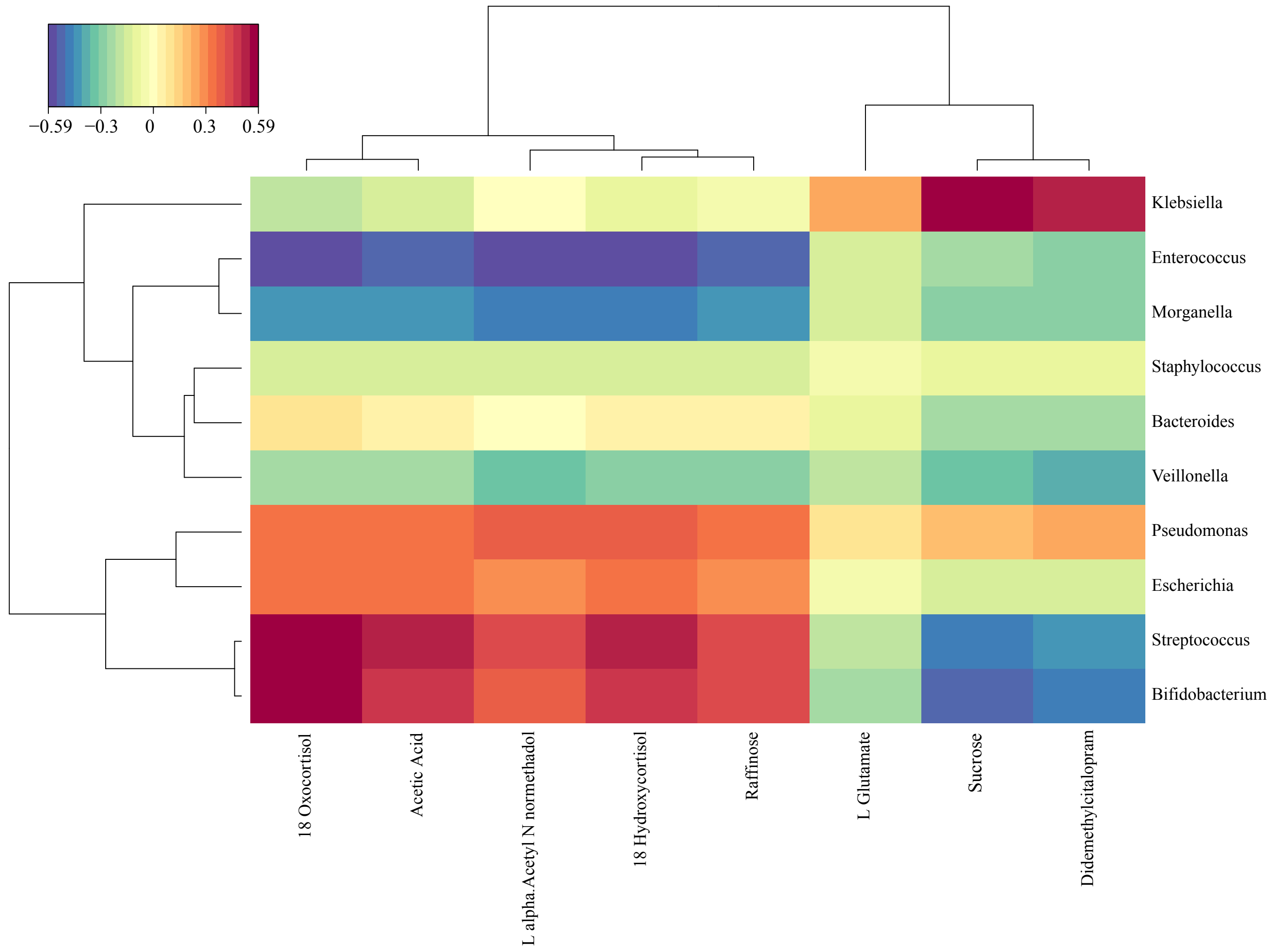

Supplement: Supplementary file 5 — Spares partial least squared correlations (sPLS) between dominant bacterial genera and identified metabolites from control infants only. Analysis excludes infants diagnosed with late onset sepsis. Only significant metabolites based on the samples at diagnosis (time point 0) and the top 10 most abundant bacterial taxa were included. (PDF 145 kb) [file 40168_2017_295_MOESM5_ESM.pdf]
