# Supplementary material for: Longitudinal development of the gut microbiome and metabolome in preterm neonates with late onset sepsis and healthy controls
Source: Microbiome. 2017 Jul 12;5:75. doi: 10.1186/s40168-017-0295-1 (PMC5508794; doi:10.1186/s40168-017-0295-1)
Supplement: Supplementary file 4 — Heatmaps of the most dominant bacterial genera per each preterm gut community type (PGCT). (A) PGCT 1. (B) PGCT 2. (C) PGCT 3. (D) PGCT 4. (E) PGCT 15. (F) PGCT 6. Genera shown in bold are dominant in that PGCT. (PDF 348 kb) [file 40168_2017_295_MOESM4_ESM.pdf]

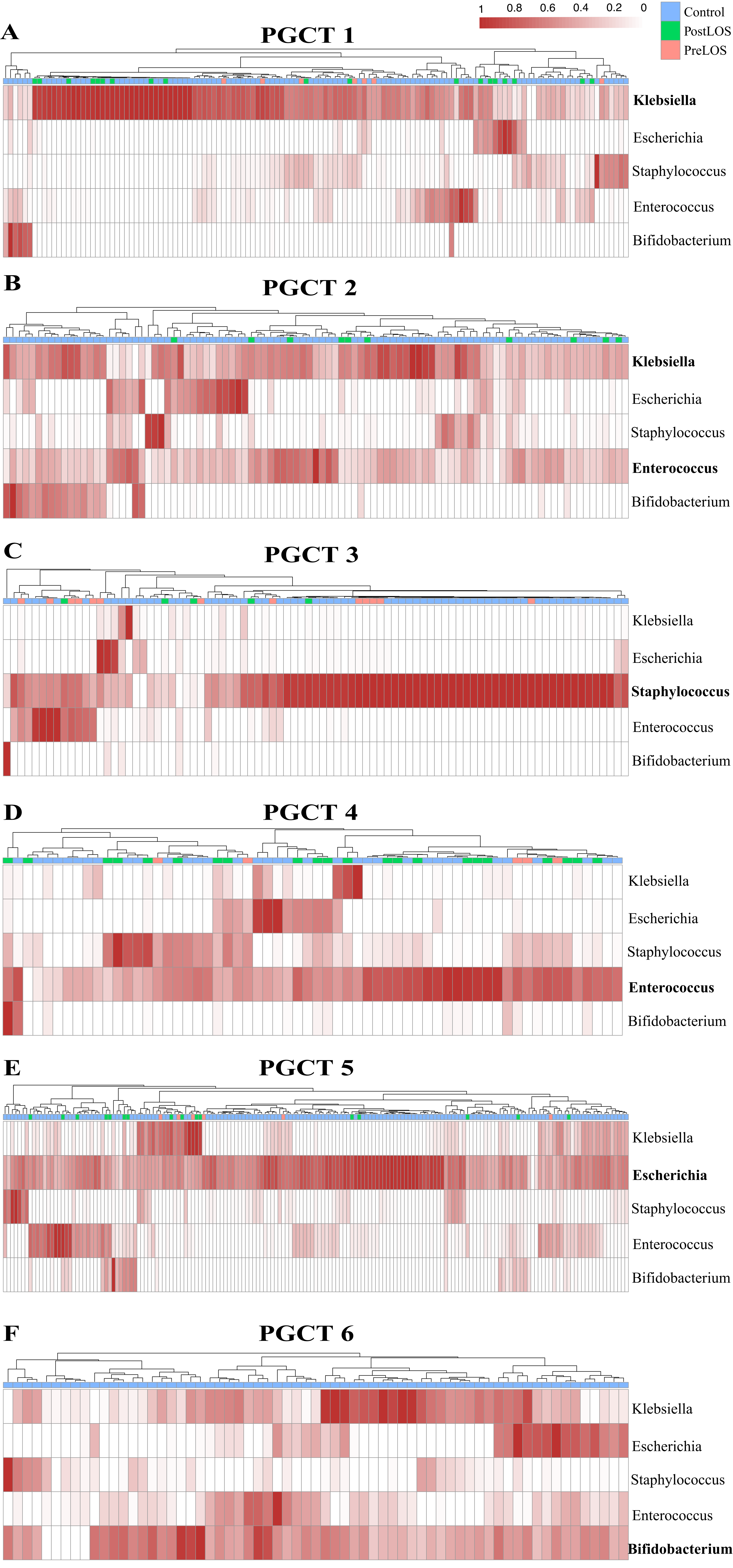

1
0.8
0.6
0.4
0.2
0
Control
PostLOS
PreLOS
A
PGCT 1
Klebsiella
Escherichia
Staphylococcus
Enterococcus
Bifidobacterium
B
PGCT 2
Klebsiella
Escherichia
Staphylococcus
Enterococcus
Bifidobacterium
C
PGCT 3
Klebsiella
Escherichia
Staphylococcus
Enterococcus
Bifidobacterium
D
PGCT 4
Klebsiella
Escherichia
Staphylococcus
Enterococcus
Bifidobacterium
E
PGCT 5
Klebsiella
Escherichia
Staphylococcus
Enterococcus
Bifidobacterium
F
PGCT 6
Klebsiella
Escherichia
Staphylococcus
Enterococcus
Bifidobacterium
